# Stratified predictions of upper limb motor outcomes after stroke

**DOI:** 10.3389/fneur.2023.1323529

**Published:** 2024-01-04

**Authors:** Chang-hyun Park, Min-Su Kim

**Affiliations:** ^1^Division of Artificial Intelligence and Software, College of Engineering, Ewha Womans University, Seoul, Republic of Korea; ^2^Department of Physical Medicine and Rehabilitation, Soonchunhyang University Cheonan Hospital, Cheonan, Republic of Korea; ^3^Department of Regenerative Medicine, College of Medicine, Soonchunhyang University, Cheonan, Republic of Korea

**Keywords:** upper limb motor impairment, upper limb motor outcome, brain structural damage, proportional recovery, predictive model

## Abstract

**Introduction:**

Longitudinal observations of upper limb motor recovery after stroke have suggested that certain subgroups may exhibit distinct recovery patterns. Here we sought to examine whether the predictive ability for post-stroke upper limb motor outcomes could be enhanced by applying conventional stratification strategies.

**Method:**

For 60 individuals who suffered the first stroke, upper limb motor impairment was assessed with the upper extremity Fugl-Meyer assessment (UE-FMA) at 2 weeks as a baseline and then 3 months post-stroke. Brain structural damage at baseline was assessed by MRI data-derived markers ranging from traditional lesion size to the lesion load and to the disconnectome. Linear regression models for predicting upper limb motor outcomes (UE-FMA score at 3 months post-stroke) based on baseline upper limb motor impairment (UE-FMA score at 2 weeks post-stroke), brain structural damage, and their combinations were generated, and those with the best predictive performance were determined for individual subgroups stratified according to initial impairment (severe and non-severe), lesion location (cortical and non-cortical), and neurophysiological status (motor evoked potential-positive and motor evoked potential-negative).

**Results:**

The best predictions were made by baseline upper limb motor impairment alone for subgroups with less functional impairment (non-severe) or less structural involvement (non-cortical), but by the combination of baseline upper limb motor impairment and brain structural damage for the other subgroups. The predictive models tailored for subgroups determined according to initial impairment and neurophysiological status yielded a smaller overall error than that for the whole group in upper limb motor outcome predictions.

**Discussion:**

The predictive ability for upper limb motor outcomes could be enhanced beyond the one-size-fits-all model for all individuals with stroke by applying specific stratification strategies, with stratification according to initial impairment being the most promising. We expect that predictive models tailored for individual subgroups could lead closer to the personalized prognosis of upper limb motor outcomes after stroke.

## 1 Introduction

Numerous investigations have attempted to understand patterns of functional recovery after stroke, yielding longitudinal observations that have revealed recovery patterns regarding the time-dependency of functional recovery. With respect to the understanding of post-stroke recovery patterns, there appear to be some concerns; among others, identifying distinct recovery patterns to address inter-individual variability in recovery courses and linking the identified recovery patterns with markers collected at baseline to predict specific recovery patterns for individuals to follow appear to be the most pressing.

These concerns have led to the development of models enabling individualized outcome predictions. As artificial intelligence approaches become increasingly available, various machine learning algorithms ranging from linear regression to deep learning have been applied to demographic, clinical, electrophysiological, and neuroimaging data, as well as their combinations, as inputs, suggesting the potential of multidimensional markers for more accurate outcome predictions (for reviews, see (1, 2)).

Wide applications of the proportional recovery rule, notably to upper limb motor outcomes (3, 4), suggest that the severity of initial impairment affects the degree of outcomes. Considering confounders of the proportional recovery rule, however, initial impairment appears to explain a smaller amount of the variance in recovery than originally assumed (5). Furthermore, the existence of individuals not fitted to the proportional recovery rule indicates that outcomes may not be well predicted by initial impairment alone for some subgroups.

In this contribution, we employed two main strategies to generate models for predicting upper limb motor outcomes after stroke. First, as a follow-up to our previous study (6), we recognized the potential of lesion-induced brain structural damage in addition to baseline upper limb motor impairment. Among lots of markers that can be obtained from neuroimaging, we believe that lesion-induced brain structural damage could best characterize individual strokes, so we considered measures ranging from traditional lesion size to the lesion load and to the disconnectome as markers. Second, we assumed that predictive performance could be improved by generating models specific to distinct recovery patterns. Since distinct recovery patterns that best describe inter-individual variability in recovery courses remain unclear, we hypothetically considered conventional stratification strategies, such as the severity of initial impairment (7, 8), the location of lesions (9, 10), and neurophysiological status (11, 12), as potentially reflecting inter-individual variability in upper limb motor recovery.

Among the different combinations of baseline upper limb motor impairment and brain structural damage, we searched for the best predictive models for individual subgroups assigned according to the conventional stratification strategies. We sought to determine whether predictive models of upper limb motor outcomes for stratified subgroups could yield a reduction in the overall error compared with that for the whole group. We hypothesized that predictive performance could be improved for specific stratification strategies if they could at least partially reflect different recovery courses across individuals.

## 2 Methods

### 2.1 Participants

**Figure 1:**
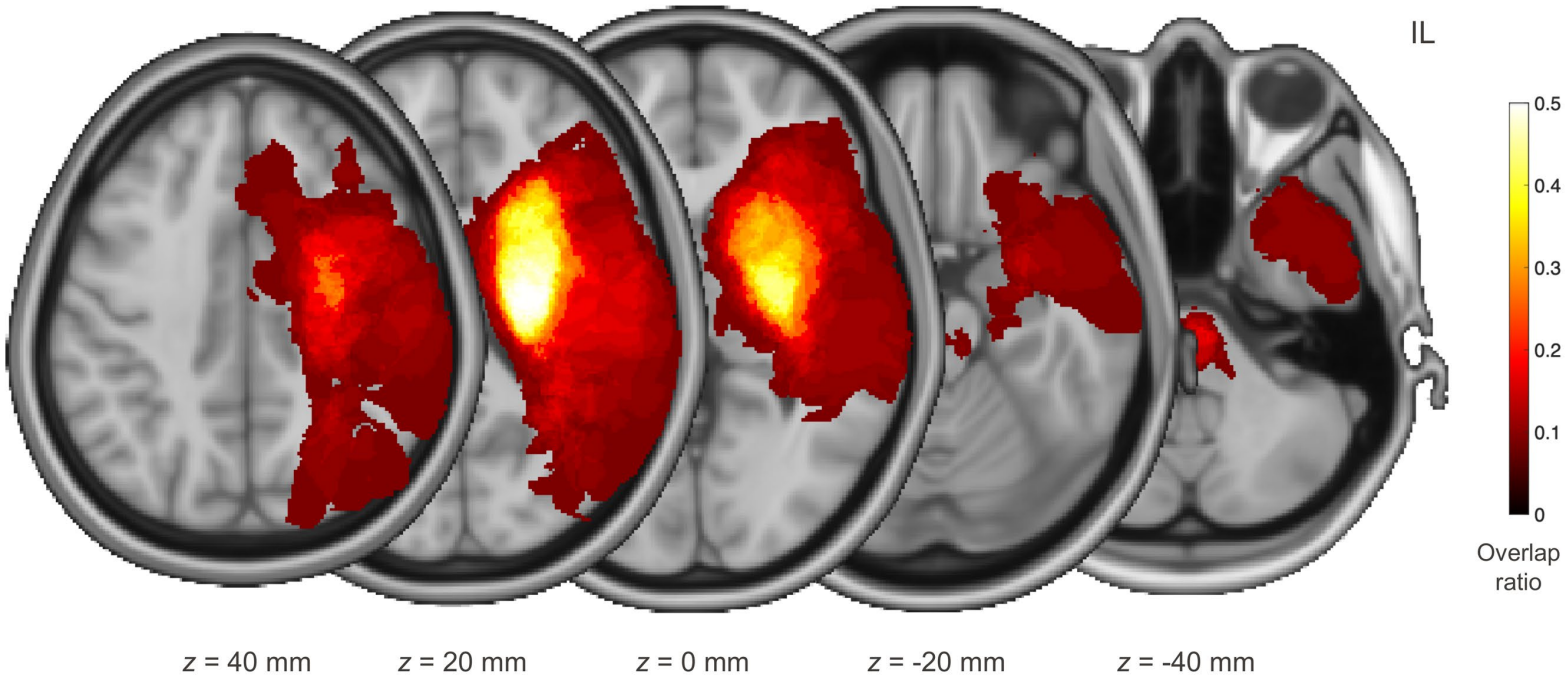
Distribution of lesions for individuals with stroke. Each individual’s lesion was transformed to the standard space, oriented to be located on the same side, and then superimposed. The red–yellow colour maps the voxel-wise overlap ratio over the individuals. IL, ipsilesional hemisphere.

Sixty individuals (59.4 ± 12.5 years, 30 women) who suffered their first stroke and had a course of disease within 2 weeks (2 W) to 3 months (3 M) post-stroke participated in this study. They included those (i) with unilateral supratentorial lesions from ischemic or hemorrhagic stroke, (ii) aged between 18 and 80 years, and (iii) who were conscious and lacked indications of dementia or mental impairment. The absence of cognitive impairment for all individuals was checked by using the Mini-Mental State Examination as a screening instrument. The individuals’ demographic and clinical characteristics are summarized in Table 1, with individual values listed in Supplementary Table S1. Lesions were manually segmented by an experienced physician, with their reliability checked by another experienced physician. An overlap map of the lesions is depicted in Figure 1. Seventy-seven healthy individuals (46.9 ± 16.5 years, 40 women) without any history of neurological or psychiatric diseases served as age- and sex-matched normative controls. Written informed consent was obtained from all participants in accordance with the Declaration of Helsinki and its later amendments, and the study was approved by the local institutional review board.

**Table 1:** Summary characteristics of participants.

			Individuals with stroke (*n* = 60)	Normative controls (*n* = 77)	Statistical comparison
Demographics	Age (years) (mean ± SD)		28 ~ 80 (59.4 ± 12.5)	22 ~ 77 (46.9 ± 16.5)	NS
	Sex		Men:women = 30:30	Men:women = 37:40	NS
Lesion side	Hemispheric motor dominance		Dominant:non-dominant = 27:33	n/a	n/a
Upper limb motor impairment	UE-FMA score (mean ± SD)	2 W	4 ~ 63 (25.2 ± 18.2)	n/a	n/a
		3 M	4 ~ 66 (39.9 ± 19.8)	n/a	n/a
Stratification	Initial impairment		Severe:non-severe = 33:27	n/a	n/a
	Lesion location		Cortical:non-cortical = 26:34	n/a	n/a
	Neurophysiological status		MEP-negative:MEP-positive = 14:33	n/a	n/a
	Proportional recovery		Non-fitted:fitted = 22:38	n/a	n/a

For individuals with stroke, the dominant/non-dominant lesion side indicates that a lesion is located in the motor dominant/non-dominant hemisphere.

UE-FMA, upper extremity Fugl-Meyer assessment; 2 W, two weeks after stroke; 3 M, three months after stroke; MEP, motor evoked potential; SD, standard deviation; and NS, non-significant.

### 2.2 Stroke rehabilitation therapy

The individuals with stroke received comprehensive rehabilitation therapy to restore motor functions from the time of study participation until 3 months post-stroke. They participated in rehabilitation therapy 5 times a week in a rehabilitation hospital, with physical and occupational therapy received twice a day, each in the morning and afternoon, for a total treatment time of 4 h per day. Physical therapy consisted of gait training, strength strengthening exercises, balance training, and joint range of motion exercises, while occupational therapy included training for activities of daily living, hand function movement exercises, and swallowing facilitation therapy. For the individuals with aphasia or dysarthria, speech therapy was added to the program.

### 2.3 Assessment of upper limb motor impairment

For the individuals with stroke who suffered motor impairment of the contralesional upper limb, the degree of upper limb motor impairment at 2 W and 3 M after stroke was assessed using the upper extremity Fugl-Meyer assessment (UE-FMA) (13) by a trained occupational therapist blinded to the individuals’ severity and not involved in administering the interventions. The UE-FMA score was based on direct observations of performance, such that each item was scored based on one’s ability to complete the item using a three-point ordinal scale (0, unable to perform; 1, partially done; and 2, fully done). The UE-FMA score at 2 W was regarded as baseline upper limb motor impairment and the UE-FMA score at 3 M was considered as upper limb motor outcomes. The average UE-FMA scores were 25.2 ± 18.2 at 2 W and 39.9 ± 19.8 at 3 M against a full score of 66.

### 2.4 Measurement of motor evoked potentials

Motor evoked potentials (MEPs) were measured for 47 of the 60 individuals; the remaining individuals were unable to meet the schedules for MEP assessment. By using a BiStim^2^ transcranial magnetic stimulation (TMS) system (Magstim, Carmarthenshire, UK) and a 70 mm figure-eight coil, a single-pulse TMS was repeatedly applied to the optimal scalp position to determine each individual’s resting motor threshold, which was defined as the lowest stimulation intensity required to produce an MEP peak-to-peak amplitude ≥50 μV in 5 of 10 consecutive trials (14). By recording MEPs via surface electrodes from the contralateral first dorsal interosseous muscle, the absence of MEPs was defined if no MEP appeared after three consecutive discharges at full power (15).

### 2.5 Acquisition and analysis of MRI data

Using an Achieva 3 T MRI system (Philips Healthcare, Best, Netherlands), structural MRI (sMRI) and diffusion-weighed MRI (dMRI) data were collected for the individuals. For sMRI data, a T1-weighted volume image was acquired in axial planes with the following parameters: number of slices = 124, slice thickness = 1.60 mm, matrix size = 512 × 512, and in-plane resolution = 0.47 mm × 0.47 mm. For dMRI data, 46 volume images comprising 45 with diffusion weighting at *b* value = 1,000 s/mm^2^ and one without diffusion weighting were acquired in axial planes with the following parameters: number of slices = 60, slice thickness = 2.25 mm, matrix size = 112 × 112, and in-plane resolution = 1.96 mm × 1.96 mm.

Preprocessing of the dMRI data was conducted using tools in FSL^1^ in such a way that eddy current-induced distortion and head movement were corrected and the skull was removed before modelling the diffusion tensor at each voxel. White matter (WM) tractography was performed for the preprocessed dMRI data to reconstruct WM fibers over the whole brain by using tools in MRtrix3.^2^ For registration between the dMRI native space and the standard space, a deformation field was estimated for the sMRI data coregistered to the dMRI data by using tools in SPM12.^3^

### 2.6 Assessment of brain structural damage

As lesion-induced brain structural damage that could reflect an effect of a lesion on the CST or whole brain, we considered three types of measures as listed in Table 2, each identified by applying a specific damage map to a region of interest (ROI). Damage maps included (i) a weighted map of fractional anisotropy (FA) (16) derived from the diffusion tensor estimated using the post-stroke individuals’ dMRI data, (ii) a binary map of a lesion identified using the post-stroke individuals’ sMRI data, and (iii) a weighted map of a structural disconnectome estimated using the normative controls’ dMRI data, as an ensemble of structural connections passing through a lesion (17, 18). Given a lesion identified for an individual with stroke, after the lesion was transformed to the dMRI data native space of a normative control, WM fibers passing through the lesion were selected among whole brain WM fibers estimated for the normative control. The distribution of voxel-wise counts of WM fibers was normalized to the maximum count and transformed to the standard space, and its average over the normative controls served as a structural disconnectome from the lesion.

**Table 2:** List of brain structural damage measures.

Type	Damage map	ROI	Structural damage measure
CST disintegrity	FA	Patient CST	Patient CST FA asymmetry
	FA	Control CST	Control CST FA asymmetry
CST damage	Lesion	Control CST	CST lesion load
	Structural disconnectome	Control CST	CST disconnectome load
Brain damage	Lesion	Whole brain	Lesion volume
	Structural disconnectome	Whole brain	Disconnectome volume

Each measure was defined by applying a specific damage map to a region of interest (ROI).

CST, corticospinal tract; and FA, fractional anisotropy.

ROIs included the (i) CST and (ii) whole brain. The territories of the CST were determined using either the post-stroke individuals’ dMRI data (patient CST) or the normative controls’ dMRI data (control CST) (19). For both patient and control CSTs, WM fibers commencing with the precentral gyrus, progressing to the posterior limb of the internal capsule (PLIC), and reaching the pons ipsilateral to the precentral gyrus were selected among whole brain WM fibers. The precentral gyrus was defined based on the respective label of the Destrieux atlas (20), and the PLIC and pons were delineated manually. Of the selected WM fibers, those extending into the cerebellum or contralateral hemisphere were excluded. The distribution of voxel-wise counts of WM fibers was normalized to the maximum count and then transformed to the standard space. The patient CST was identified for each individual with stroke, while the control CST was determined as the average of the CSTs of the normative controls.

A total of six measures of brain structural damage, including two CST disintegrity measures, two CST damage measures, and two brain damage measures, were evaluated for the individuals with stroke at baseline. CST disintegrity measures, including patient CST FA asymmetry and control CST FA asymmetry, were acquired by applying an FA map to the patient or control CST, averaging voxel-wise FA values in each hemisphere, and computing the asymmetry of the mean FA values between the two hemispheres: (FA_contralesional_ - FA_ipsilesional_)/(FA_contralesional_ + FA_ipsilesional_). CST damage measures, including the CST lesion load and CST disconnectome load, were obtained by applying a lesion or disconnectome map to the control CST and computing the weighted volume of the overlap. Brain damage measures, including lesion volume and disconnectome volume, were acquired by applying a lesion or disconnectome map to the whole brain and computing the weighted volume of the overlap. To check whether there was multicollinearity between the six measures of brain structural damage, partial correlation between them was assessed after controlling for the individuals’ age, sex, and hemispheric motor dominance.

### 2.7 Subgroup stratification

The individuals with stroke were allocated to subgroups according to (i) initial impairment, (ii) lesion location, and (iii) neurophysiological status. The severity of initial upper limb motor impairment was evaluated in terms of the UE-FMA score at 2 W. Among the 60 individuals, 33 with a score of 20 or lower were classified as severe (13), whereas the other 27 with a score higher than 20 were classified as non-severe. The criterion for the distribution of lesion location was determined according to whether lesions involved cortical areas beyond subcortical areas. Among the 60 individuals, 26 with lesions that involved both subcortical and cortical areas were classified as cortical, whereas the other 34 with lesions that involved subcortical areas only were classified as non-cortical. Neurophysiological status was assessed in terms of MEPs elicited by TMS. Among the 47 individuals for whom MEPs were evaluated, 14 without MEPs were classified as MEP-negative, whereas the other 33 with MEPs recorded in either contralateral target muscle were classified as MEP-positive.

For comparison with the stratification strategies considered above, the individuals were also assigned to subgroups according to whether they met the proportional recovery rule. Defining a model residual as the difference between the predicted change in the UE-FMA score according to the proportional recovery rule ((66 - UE-FMA score at 2 W) × 0.7) and the observed change in the UE-FMA score (UE-FMA score at 3 M - UE-FMA score at 2 W), larger model residuals indicated recovery-atypical individuals showing much poorer upper limb motor outcomes than predicted by the proportional recovery rule (21). Among the 60 individuals, 22 with a model residual of 20 or larger were classified as non-fitted (21), whereas the other 38 with a model residual smaller than 20 were classified as fitted, as displayed in the histogram of model residuals in Fig. S1.

### 2.8 Construction and comparison of predictive models

We used multiple linear regression to generate models for predicting upper limb motor outcomes. That is, upper limb motor outcomes served as the response variable and inputs including baseline upper limb motor impairment and brain structural damage served as predictor variables. Specifically, three kinds of models were generated according to inputs employed: (i) baseline upper limb motor impairment alone, (ii) brain structural damage alone, and (iii) a combination of baseline upper limb motor impairment and brain structural damage. In all the models, the individuals’ age, sex, and hemispheric motor dominance were included as confounding covariates. Model parameters were estimated by using the least square approximation without involving regularization. This model construction was repeated for the whole group and for each stratified subgroup.

To assess the predictive ability of each model, the in-sample error and the out-of-sample or generalization error in predicting upper limb motor outcomes were measured by the squared error per sample, that is, the mean squared error (MSE). While the in-sample error was estimated for samples already seen in the training phase, the generalization error was estimated for those unseen in the training phase. Specifically, the generalization error was measured via leave-one-out cross-validation (CV), in which a squared error was computed for each left-out sample when the remaining samples were used to estimate the model parameters. A model’s superiority was primarily determined by a smaller MSE in CV in terms of the generalization error since the robustness of a model to a range of unseen samples beyond those used for estimating model parameters would be crucial for assessing the model’s practical applicability. Moreover, the goodness of fit of each model was evaluated by the coefficient of determination, *R*^2^, and the trade-off between the goodness of fit and model complexity was evaluated by the corrected Akaike information criterion (AICc) (22).

Between nested models, specifically between a reduced model composed of baseline upper limb motor impairment alone and an extended model comprising a combination of baseline upper limb motor impairment and brain structural damage, the likelihood-ratio *χ*^2^ test was carried out to assess whether adding brain structural damage as an additional predictor variable could significantly improve the goodness of fit. In addition, between different models formed by a combination of baseline upper limb motor impairment and brain structural damage, a difference in the goodness of fit was evaluated by comparing *R*^2^ values. In all statistical inferences, statistical significance was identified when a *p* value was less than 0.05, specifically corrected for multiple comparisons by a false discovery rate approach in the case of considering multiple models.

## 3 Results

### 3.1 Correlation between brain structural damage measures

The six measures of brain structural damage were highly correlated with each other (*p* < 0.001 for all pairs), with correlation coefficients ranging from 0.525 to 0.920 (Fig. S2). The average correlation coefficients between each and the others were 0.766, 0.803, 0.839, 0.698, 0.727, and 0.837 for the measures as ordered in Table 2, showing that the CST lesion load was the most correlated, whereas the CST disconnectome load was the least correlated on average. Specifically, the correlation coefficient was 0.920 between the two CST disintegrity measures, 0.809 between the two CST damage measures, and 0.862 between the two brain damage measures.

### 3.2 Predictive models for stratified subgroups

Since generally high correlation between the six measures of brain structural damage indicated multicollinearity between them, each of the measures was individually employed as a predictor variable, producing a total of 13 multiple linear regression models, as listed in Supplementary Table S2, for predicting upper limb motor outcomes. The statistics of the models are listed in Supplementary Table S3. In all models constructed, baseline upper limb motor impairment was a statistically significant predictor when it was combined with brain structural damage as well as when it was employed alone. When baseline upper limb motor impairment was combined with brain structural damage, brain structural damage was a statistically significant predictor for specific subgroups and, in connection with that, adding brain structural damage to baseline upper limb motor impairment could offer, but not always, a significant improvement in the goodness of fit. In addition, between the models comprised of a combination of baseline upper limb motor impairment and brain structural damage, the goodness of fit was not significantly different as specified in Supplementary Table S4.

The best predictive models developed for the whole group and for subgroups specified according to the different stratification strategies are summarized in Table 3. While the combination of baseline upper limb motor impairment and the CST lesion load (B + LL) composed the best predictive model with the greatest *R*^2^ (*R*^2^ = 0.672) and the smallest AICc (AICc = 474.253) as well as the smallest MSE in CV (MSE = 152.572) for the whole group, the best predictive models were variable between stratified subgroups. Whereas the best predictive models consisted of baseline upper limb motor impairment alone (B) in the non-severe subgroup determined by initial impairment and in the non-cortical subgroup determined by lesion location, the combination of baseline upper limb motor impairment and the CST disconnectome load (B + DL) formed the best predictive model for the severe subgroup determined by initial impairment, for the cortical subgroup determined by lesion location, and for the MEP-negative subgroup determined by neurophysiological status.

**Table 3:** Predictive ability of the best predictive models developed for the whole group and for stratified subgroups.

Subgroup		Generalization error			In-sample error		
		Best model	MSE	Overall MSE	Best model	MSE	Overall MSE
All individuals		B + LL	152.572	152.572 (100%)	B + LL	140.510	140.510 (100%)
Initial impairment	Severe	B + DL	149.457	130.982 (85.8%)	B + DL	122.040	106.068 (75.5%)
	Non-severe	B	108.402		B + PF	86.547	
Lesion location	Cortical	B + DL	193.887	162.572 (106.6%)	B + DL	161.464	135.831 (96.7%)
	Non-cortical	B	138.802		B + DV	116.230	
Neurophysiological status	MEP-negative	B + DL	156.777	112.877 (96.1%)	B + DL	128.301	92.011 (89.6%)
	MEP-positive	B + DV	9.398		B + DV	6.470	
Proportional recovery	Non-fitted	B + PF	43.753	46.434 (30.4%)	B + PF	31.732	37.648 (26.8%)
	Fitted	B	47.986		B + DL	41.073	

A smaller mean squared error (MSE) indicates better predictive ability.

CV, cross-validation; MEP, motor evoked potential; B, baseline upper extremity Fugl-Meyer assessment (UE-FMA) score; B + PF, baseline UE-FMA score + patient CST FA asymmetry; B + LL, baseline UE-FMA score + CST lesion load; B + DL, baseline UE-FMA score + CST disconnectome load; and B + DV, baseline UE-FMA score + disconnectome volume.

### 3.3 Overall error of predictive models for stratified subgroups

In predicting upper limb motor outcomes, having set the MSE of the best predictive model generated for the whole group at 100%, the best predictive models constructed via subgroup stratification according to initial impairment, lesion location, and neurophysiological status yielded overall MSEs ranging from 85.8 to 106.6% for the generalization error and those ranging from 75.5 to 96.7% for the in-sample error, as listed in Table 3, when the overall MSE was computed by weighting the MSEs of predictive models for stratified subgroups by the number of individuals in each subgroup. By comparison, for subgroups determined according to proportional recovery, as reference for those exhibiting different recovery courses, the best predictive model yielded an overall MSE reduced up to 30.4% for the generalization error and up to 26.8% for the in-sample error.

## 4 Discussion

In predicting upper limb motor outcomes in stroke recovery, we showed that predictive models tailored for subgroups of individuals with stroke could be furnished by applying conventional stratification strategies. While baseline upper limb motor impairment alone composed better predictive models for the non-severe subgroup determined by initial impairment and the non-cortical subgroup determined by lesion location, a combination of baseline upper limb motor impairment and brain structural damage formed superior predictive models for the other subgroups as well as for the whole group. We demonstrated that predictive models tailored for subgroups based on specific stratification strategies, such as initial impairment and neurophysiological status, could lead to reductions in the overall error in upper limb motor outcome predictions compared with the predictive model for the whole group.

An increasing number of studies of upper limb motor recovery after stroke have suggested numerous markers sourced from various clinical data, not least of which are upper limb motor impairment and neuroimaging-based brain structural damage evaluated at baseline (23), in predicting subsequent upper limb motor outcomes. While initial impairment is a well-known marker of outcomes several months later (24, 25), we showed that a combination of baseline upper limb motor impairment and brain structural damage could generally provide an improvement in predictive ability compared with the use of baseline upper limb motor impairment alone. Considering the relevance of proportional recovery to lesion-induced CST disintegrity (3, 21, 26) and CST damage (7), brain structural damage appears to have the potential to improve the predictive ability when used together with baseline upper limb motor impairment (6).

For the non-severe subgroup determined by initial impairment and the non-cortical subgroup determined by lesion location, upper limb motor outcomes were best predicted by baseline upper limb motor impairment alone, specifically in terms of the generalization error. Considering that variability in recovery courses could be associated with the heterogeneity of lesion characteristics between individuals (9, 10, 27–29), structurally or functionally less impairment would induce smaller inter-individual variability; hence, the greatest robustness to variations in upper limb motor outcome predictions could be achieved by the simpler predictive model composed of baseline upper limb motor impairment alone for the subgroups.

As the virtue of upper limb motor outcome predictions for stratified subgroups, we showed that predictive models tailored for subgroups determined according to specific stratification strategies could yield a smaller overall error compared with that for the so called one-size-fits-all model for all individuals with stroke. Of the stratification strategies considered here, subgroup stratification according to initial impairment appears to be most promising in that the predictive models for the stratified subgroups yielded smaller MSEs in terms of the generalization error for every subgroup than that for the one-size-fits-all model. Considering that many individuals not fitted to the proportional recovery rule are those with initially severe impairment (30), subgroup stratification according to initial impairment appears to partly reflect different recovery courses as implied by proportional recovery. In subgroup stratification according to neurophysiological status, the MSE in terms of the generalization error was greatly reduced for the MEP-positive subgroup, but not for the MEP-negative subgroup, indicating much larger variability in recovery courses in the absence of MEPs (31) in contrast to the robust predictive value in the presence of MEPs (12).

In upper limb motor recovery studies that considered follow-up outcomes as the response variable to be predicted, differing ability in outcome predictions according to subgroups has been often presented. For example, Feng and colleagues (7) showed that baseline upper limb motor impairment was a marker comparable to the CST lesion load in predicting upper limb motor outcomes for the whole sample, but not for a subgroup with initially severe impairment (UE-FMA score at baseline ≤ 10). Such changing contributions of a certain marker to predicting outcomes appear to suggest a need for predictive models for stratified subgroups as a possible way to improve predictive performance, as well as supporting the notion of grossly different recovery patterns across individuals. In this respect, we note that our unique attempt was to generate predictive models for individual subgroups assigned according to hypothetical differences in recovery patterns, while many of previous studies aimed to predict different recovery patterns themselves via the representation of the response variable by categories of outcomes, for example, two recovery patterns in the proportional recovery rule (32) and four recovery patterns in the Predict Recovery Potential algorithm (33). Our approach may find new applications as distinct recovery patterns that better describe inter-patient variability in recovery courses could be identified in the future.

This study has some limitations that should be accounted for in future studies. First, the predictive models suggested here are not yet considered conclusive primarily due to the small sample size. Subgroup stratification according to initial impairment appears to be a reasonable starting point for further development of predictive models for stratified subgroups, but it would be necessary to refine the stratification strategy and validate the performance of the predictive models against a larger sample for practical application to prognostic predictions in clinical practice. Second, although here we considered at most two subgroups for stratification strategies partly because of the limited pool of post-stroke individuals, the number of subgroups could vary greatly. For instance, by considering that the presence of MEPs could be a useful marker particularly for individuals with initially severe impairment (12), initial impairment and neurophysiological status might be applied together to provide subdivided subgroups according to a combination of stratification strategies. Third, more markers than those considered here could be added to establish more robust and accurate outcome predictions. High-dimensional markers from demographic, clinical, electrophysiological, and neuroimaging data would increase the opportunity to apply more complex artificial intelligence approaches and eventually render individualized outcome predictions practically feasible.

## 5 Conclusion

Despite growing momentum to develop precision medicine for stroke, the phenotypic diversity of stroke appears to be a main challenge specifically in predicting functional outcomes after stroke. In the current study, we put forward the value of subgroup stratification in developing prognostic predictive models of upper limb motor outcomes. We suppose that predictive models for stratified subgroups could serve as an intermediate step towards more complete precision medicine for personalized prognosis of upper limb motor outcomes, thus paving the way for promoting clinical application of such prognostic predictive models in stroke recovery.

## Data availability statement

The raw data supporting the conclusions of this article will be made available by the authors, without undue reservation.

## Ethics statement

This studies involving humans were approved by the Institutional Review Board of Soonchunhyang University Cheonan Hospital (No. 2022-07-084-001). The studies were conducted in accordance with the local legislation and institutional requirements. The participants provided their written informed consent to participate in this study.

## Author contributions

ChP: Conceptualization, Data curation, Formal analysis, Funding acquisition, Investigation, Methodology¸ Project administration, Resources, Software, Supervision, Validation, Visualization, Writing – original draft, Writing – review & editing. M-SK: Conceptualization, Funding acquisition, Investigation, Project administration, Writing – review & editing.

## Footnotes

^1^ https://fsl.fmrib.ox.ac.uk/

^2^ https://www.mrtrix.org/

^3^ http://www.fil.ion.ucl.ac.uk/spm/
