# Supplementary material for: Stratified predictions of upper limb motor outcomes after stroke
Source: Front Neurol. 2024 Jan 4;14:1323529. doi: 10.3389/fneur.2023.1323529 (PMC10794733; doi:10.3389/fneur.2023.1323529)
Supplement: Supplementary file 1 [file Data_Sheet_1.PDF]

Supplementary Material:

Stratified predictions of upper limb motor outcomes after stroke

Chang-hyun Park[1], Min Su Kim[2,3]

[1]Division of Artificial Intelligence and Software, College of Engineering, Ewha Womans University, Seoul, Korea
[2]Department of Physical Medicine and Rehabilitation, Soonchunhyang University Cheonan Hospital, Cheonan, Korea
[3]Department of Regenerative Medicine, College of Medicine, Soonchunhyang University, Cheonan, Korea

Contents:

• Supplementary Tables: Tables S1-S4
• Supplementary Figures: Figs. S1-S2

Supplementary Tables

Table S1. Demographic and clinical characteristics of individuals with stroke. Hemispheric motor dominance is 1 when the motor dominant hemisphere agrees with the ipsilesional hemisphere and 0 otherwise.

No	Age (years)	Sex	Hemispheric dominance	UE-FMA score		Proportional recovery	Lesion location	MEP response
				2 W	3 M			
1	33	M	1	41	62	Fitter	SC	Absence
2	63	M	1	24	21	Nonfitter	SC	Absence
3	60	M	0	29	46	Fitter	C+SC	Absence
4	60	M	0	10	14	Nonfitter	C+SC	n/a
5	79	W	0	20	47	Fitter	C+SC	n/a
6	48	W	0	18	41	Fitter	SC	Absence
7	71	W	0	34	58	Fitter	SC	Presence
8	53	W	0	58	60	Fitter	SC	Presence
9	75	M	0	60	58	Fitter	C+SC	Presence
10	66	M	0	44	55	Fitter	C+SC	Presence
11	65	W	0	55	66	Fitter	SC	Presence
12	66	M	1	62	66	Fitter	C+SC	Presence
13	55	M	1	56	62	Fitter	C+SC	Presence
14	60	W	1	45	59	Fitter	SC	Absence
15	67	W	1	28	57	Fitter	SC	Presence
16	70	M	1	50	66	Fitter	SC	Absence
17	66	W	1	12	20	Nonfitter	SC	n/a
18	61	W	1	5	7	Nonfitter	C+SC	Absence
19	55	W	0	20	45	Fitter	SC	Presence
20	54	M	0	49	57	Fitter	C+SC	Presence
21	54	W	0	63	65	Fitter	SC	Presence
22	52	M	1	27	50	Fitter	SC	Absence
23	28	W	0	5	12	Nonfitter	C+SC	Absence
24	58	W	1	10	27	Nonfitter	SC	Absence
25	39	M	0	48	66	Fitter	SC	Absence
26	53	M	1	5	44	Fitter	SC	Absence
27	47	W	0	17	59	Fitter	C+SC	n/a
28	55	M	1	10	43	Fitter	C+SC	Presence
29	37	W	1	11	60	Fitter	C+SC	Absence
30	61	W	0	15	18	Nonfitter	C+SC	n/a
31	52	M	1	30	55	Fitter	C+SC	n/a
32	60	M	0	9	9	Nonfitter	C+SC	Absence
33	52	W	0	18	34	Fitter	SC	Absence
34	67	M	0	6	20	Nonfitter	SC	Absence
35	35	W	1	6	14	Nonfitter	C+SC	n/a
36	68	M	0	22	58	Fitter	C+SC	n/a
37	42	W	1	4	20	Nonfitter	C+SC	Absence
38	32	M	1	6	37	Fitter	C+SC	Presence
39	45	W	0	15	47	Fitter	SC	Absence
40	33	M	0	16	37	Fitter	SC	Absence
41	69	W	0	11	11	Nonfitter	SC	Absence
42	69	W	1	17	40	Fitter	C+SC	Absence
43	49	M	0	17	36	Fitter	SC	Absence
44	67	W	0	18	18	Nonfitter	SC	Absence
45	52	W	0	16	16	Nonfitter	SC	n/a
46	55	W	1	36	36	Nonfitter	SC	Absence
47	58	W	1	4	4	Nonfitter	C+SC	n/a
48	64	M	1	10	24	Nonfitter	C+SC	Absence
49	68	M	0	15	20	Nonfitter	SC	Absence
50	69	M	1	28	30	Nonfitter	SC	Absence
51	69	M	0	49	66	Fitter	SC	Absence
52	79	W	0	8	24	Nonfitter	SC	n/a

53	62	M	0	32	44	Fitter	SC	n/a
54	77	M	0	56	55	Fitter	SC	Presence
55	70	W	0	49	55	Fitter	C+SC	n/a
56	80	W	1	41	54	Fitter	SC	Absence
57	67	M	1	4	4	Nonfitter	C+SC	Absence
58	56	M	0	4	4	Nonfitter	C+SC	Absence
59	73	W	1	21	55	Fitter	SC	Absence
60	54	M	1	14	53	Fitter	SC	Absence

UE-FMA, Upper extremity Fugl-Meyer assessment; 2 W, two weeks post-stroke; 3 M, three months post-stroke; MEP, motor evoked potential; W, woman; M, man; C, cortical; and SC, subcortical.

Table S2. List of predictive models and their constituent predictor variables.

Model	Predictor variable
B	Baseline UE-FMA score
PF	Patient CST FA asymmetry
CF	Control CST FA asymmetry
LL	CST lesion load
DL	CST disconnectome load
LV	Lesion volume
DV	Disconnectome volume
B+PF	Baseline UE-FMA score<br>Patient CST FA asymmetry
B+CF	Baseline UE-FMA score<br>Control CST FA asymmetry
B+LL	Baseline UE-FMA score<br>CST lesion load
B+DL	Baseline UE-FMA score<br>CST disconnectome load
B+LV	Baseline UE-FMA score<br>Lesion volume
B+DV	Baseline UE-FMA score<br>Disconnectome volume

UE-FMA, Upper extremity Fugl-Meyer assessment; CST, corticospinal tract; and FA, fractional anisotropy.

Table S3. Statistics of predictive models developed for the whole group (A) and for stratified subgroups (B-E). The labels of the models refer to those listed in Table S2. A smaller mean squares error (MSE), a larger R^2, and a smaller corrected Akaike information criterion (AICc) indicate better predictive ability. In case of statistical significance in the F test for a linear regression model relative to the constant model, the likelihood-ratio χ^2 test for the addition of a predictor variable to the reduced model composed of baseline upper limb motor impairment alone, and the t test for a single predictor variable, respective statistics were expressed in bold.

(A) Predictive models constructed for the whole group

Patient subgroup	Model	Model statistic						Predictor variable statistic	
		MSE in CV	MSE	R^2	AICc	Significance	Comparison	Predictor variable	Significance
All	B	171.483	158.656	0.623	480.167	**$F = 22.684$ ($p < 0.001$)**	n/a	Baseline UE-FMA score	**$t = 9.405$ ($p < 0.001$)**
	PF	392.120	362.310	0.138	529.713	$F = 2.204$ ($p = 0.080$)	n/a	Patient CST FA asymmetry	**$t = -2.796$ ($p = 0.007$)**
	CF	415.822	382.801	0.089	533.014	$F = 1.350$ ($p = 0.263$)	n/a	Control CST FA asymmetry	**$t = -2.111$ ($p = 0.039$)**
	LL	369.000	344.288	0.181	526.652	**$F = 3.039$ ($p = 0.025$)**	n/a	CST lesion load	**$t = -3.333$ ($p = 0.002$)**
	DL	426.796	391.055	0.070	534.294	$F = 1.032$ ($p = 0.399$)	n/a	CST disconnectome load	$t = -1.789$ ($p = 0.079$)
	LV	381.097	357.937	0.149	528.985	$F = 2.399$ ($p = 0.061$)	n/a	Lesion volume	**$t = -2.930$ ($p = 0.005$)**
	DV	407.829	375.988	0.106	531.936	$F = 1.624$ ($p = 0.181$)	n/a	Disconnectome volume	**$t = -2.352$ ($p = 0.022$)**
	B+PF	163.312	149.748	0.650	478.073	**$F = 20.081$ ($p < 0.001$)**	**$\chi2 = 4.568$ ($p = 0.033$)**	Baseline UE-FMA score	**$t = 8.892$ ($p < 0.001$)**
								Patient CST FA asymmetry	**$t = -2.067$ ($p = 0.044$)**
	B+CF	163.862	150.005	0.650	478.176	**$F = 20.028$ ($p < 0.001$)**	**$\chi2 = 4.465$ ($p = 0.035$)**	Baseline UE-FMA score	**$t = 9.293$ ($p < 0.001$)**
								Control CST FA asymmetry	**$t = -2.043$ ($p = 0.046$)**
	B+LL	152.572	140.510	0.672	474.253	**$F = 22.111$ ($p < 0.001$)**	**$\chi2 = 8.388$ ($p = 0.004$)**	Baseline UE-FMA score	**$t = 8.987$ ($p < 0.001$)**
								CST lesion load	**$t = -2.847$ ($p = 0.006$)**
	B+DL	155.219	140.677	0.671	474.324	**$F = 22.072$ ($p < 0.001$)**	**$\chi2 = 8.317$ ($p = 0.004$)**	Baseline UE-FMA score	**$t = 9.944$ ($p < 0.001$)**
								CST disconnectome load	**$t = -2.834$ ($p = 0.006$)**
	B+LV	161.026	149.747	0.650	478.073	**$F = 20.081$ ($p < 0.001$)**	**$\chi2 = 4.569$ ($p = 0.033$)**	Baseline UE-FMA score	**$t = 8.801$ ($p < 0.001$)**
								Lesion volume	**$t = -2.067$ ($p = 0.044$)**
	B+DV	160.003	146.607	0.658	476.801	**$F = 20.742$ ($p < 0.001$)**	**$\chi2 = 5.840$ ($p = 0.016$)**	Baseline UE-FMA score	**$t = 9.330$ ($p < 0.001$)**
								Disconnectome volume	**$t = -2.350$ ($p = 0.022$)**

(B) Predictive models constructed via subgroup stratification according to initial impairment

Patient subgroup	Model	Model statistic						Predictor variable statistic	
		MSE in CV	MSE	R^2	AICc	Significance	Comparison	Predictor variable	Significance
Severe	B	170.572	144.349	0.539	264.534	**$F = 8.189$ ($p < 0.001$)**	n/a	Baseline UE-FMA score	**$t = 5.385$ ($p < 0.001$)**
	PF	253.513	213.960	0.317	277.521	**$F = 3.247$ ($p = 0.026$)**	n/a	Patient CST FA asymmetry	**$t = -3.234$ ($p = 0.003$)**
	CF	265.771	225.123	0.281	279.199	**$F = 2.739$ ($p = 0.048$)**	n/a	Control CST FA asymmetry	**$t = -2.924$ ($p = 0.007$)**

	LL	195.145	169.370	0.459	269.809	**F = 5.945 (p = 0.001)**	n/a	CST lesion load	**t = -4.537 (p < 0.001)**
	DL	238.108	203.336	0.351	275.841	**F = 3.783 (p = 0.014)**	n/a	CST disconnectome load	**t = -3.531 (p = 0.001)**
	LV	272.670	221.853	0.292	278.717	**F = 2.883 (p = 0.041)**	n/a	Lesion volume	**t = -3.015 (p = 0.005)**
	DV	258.669	219.952	0.298	278.433	**F = 2.968 (p = 0.037)**	n/a	Disconnectome volume	**t = -3.067 (p = 0.005)**
	B+PF	161.832	134.337	0.586	263.970	**F = 7.657 (p < 0.001)**	$\chi 2$ = 3.572 (p = 0.059)	Baseline UE-FMA score<br>Patient CST FA asymmetry	**t = 4.195 (p < 0.001)**<br>t = -1.757 (p = 0.090)
	B+CF	170.789	140.535	0.567	265.459	**F = 7.081 (p < 0.001)**	$\chi 2$ = 2.084 (p = 0.149)	Baseline UE-FMA score<br>Control CST FA asymmetry	**t = 4.225 (p < 0.001)**<br>t = -1.327 (p = 0.196)
	B+LL	159.461	129.734	0.601	262.820	**F = 8.120 (p < 0.001)**	$\chi 2$ = 4.723 (p = 0.030)	Baseline UE-FMA score<br>CST lesion load	**t = 3.091 (p = 0.005)**<br>t = -2.038 (p = 0.051)
	B+DL	149.457	122.040	0.624	260.802	**F = 8.973 (p < 0.001)**	$\chi 2$ = 6.740 (p = 0.009)	Baseline UE-FMA score<br>CST disconnectome load	**t = 4.433 (p < 0.001)**<br>**t = -2.474 (p = 0.020)**
	B+LV	181.068	146.243	0.550	266.773	**F = 6.594 (p < 0.001)**	$\chi 2$ = 0.770 (p = 0.380)	Baseline UE-FMA score<br>Lesion volume	**t = 3.934 (p = 0.001)**<br>t = -0.798 (p = 0.432)
	B+DV	178.958	140.591	0.567	265.472	**F = 7.076 (p < 0.001)**	$\chi 2$ = 2.071 (p = 0.150)	Baseline UE-FMA score<br>Disconnectome volume	**t = 4.099 (p < 0.001)**<br>t = -1.322 (p = 0.197)
Non-severe	B	108.402	89.794	0.392	205.383	F = 3.545 (p = 0.022)	n/a	Baseline UE-FMA score	**t = 3.166 (p = 0.004)**
	PF	144.579	118.154	0.200	212.794	F = 1.374 (p = 0.275)	n/a	Patient CST FA asymmetry	t = 1.529 (p = 0.140)
	CF	146.793	124.598	0.156	214.228	F = 1.018 (p = 0.420)	n/a	Control CST FA asymmetry	t = 1.039 (p = 0.310)
	LL	157.605	130.067	0.119	215.388	F = 0.744 (p = 0.572)	n/a	CST lesion load	t = 0.331 (p = 0.744)
	DL	152.857	130.189	0.118	215.413	F = 0.738 (p = 0.576)	n/a	CST disconnectome load	t = 0.298 (p = 0.769)
	LV	167.163	129.729	0.121	215.317	F = 0.760 (p = 0.562)	n/a	Lesion volume	t = -0.409 (p = 0.687)
	DV	155.948	130.384	0.117	215.453	F = 0.729 (p = 0.582)	n/a	Disconnectome volume	t = 0.236 (p = 0.816)
	B+PF	110.349	86.547	0.441	206.476	F = 3.307 (p = 0.023)	$\chi 2$ = 2.251 (p = 0.134)	Baseline UE-FMA score<br>Patient CST FA asymmetry	**t = 3.006 (p = 0.007)**<br>t = 1.351 (p = 0.191)
	B+CF	115.577	91.839	0.406	208.078	F = 2.875 (p = 0.039)	$\chi 2$ = 0.648 (p = 0.421)	Baseline UE-FMA score<br>Control CST FA asymmetry	**t = 2.974 (p = 0.007)**<br>t = 0.714 (p = 0.483)
	B+LL	116.081	92.205	0.404	208.186	F = 2.847 (p = 0.041)	$\chi 2$ = 0.541 (p = 0.462)	Baseline UE-FMA score<br>CST lesion load	**t = 3.168 (p = 0.005)**<br>t = 0.652 (p = 0.522)
	B+DL	119.517	94.031	0.392	208.715	F = 2.710 (p = 0.048)	$\chi 2$ = 0.011 (p = 0.915)	Baseline UE-FMA score<br>CST disconnectome load	**t = 3.076 (p = 0.006)**<br>t = -0.094 (p = 0.926)

	B+LV	116.825	94.068	0.392	208.726	F = 2.707<br>(p = 0.049)	χ2 = 0.001<br>(p = 0.979)	Baseline UE-FMA score<br>Lesion volume	**t = 3.056**<br>(**p = 0.006**)<br>t = 0.024<br>(p = 0.981)
	B+DV	115.581	94.067	0.392	208.725	F = 2.707<br>(p = 0.049)	χ2 = 0.001<br>(p = 0.974)	Baseline UE-FMA score<br>Disconnectome volume	**t = 3.081**<br>(**p = 0.006**)<br>t = -0.028<br>(p = 0.978)

(C) Predictive models constructed via subgroup stratification according to lesion location

**Patient subgroup**	**Model**	**Model statistic**						**Predictor variable statistic**	
		**MSE in CV**	**MSE**	**R^2**	**AICc**	**Significance**	**Comparison**	**Predictor variable**	**Significance**
Cortical	B	283.289	229.460	0.610	222.561	**F = 8.225**<br>(**p < 0.001**)	n/a	Baseline UE-FMA score	**t = 5.427**<br>(**p < 0.001**)
	PF	448.464	379.043	0.356	235.611	F = 2.907<br>(p = 0.046)	n/a	Patient CST FA asymmetry	**t = -3.089**<br>(**p = 0.006**)
	CF	529.167	449.884	0.236	240.066	F = 1.623<br>(p = 0.206)	n/a	Control CST FA asymmetry	**t = -2.176**<br>(**p = 0.041**)
	LL	372.026	332.404	0.436	232.197	**F = 4.052**<br>(**p = 0.014**)	n/a	CST lesion load	**t = -3.719**<br>(**p = 0.001**)
	DL	465.999	416.333	0.293	238.051	F = 2.176<br>(p = 0.107)	n/a	CST disconnectome load	**t = -2.609**<br>(**p = 0.016**)
	LV	469.577	410.903	0.302	237.709	F = 2.275<br>(p = 0.095)	n/a	Lesion volume	**t = -2.679**<br>(**p = 0.014**)
	DV	454.679	404.697	0.313	237.313	F = 2.390<br>(p = 0.083)	n/a	Disconnectome volume	**t = -2.758**<br>(**p = 0.012**)
	B+PF	250.786	197.291	0.681	220.786	**F = 8.537**<br>(**p < 0.001**)	**χ2 = 5.196**<br>(**p = 0.023**)	Baseline UE-FMA score<br>Patient CST FA asymmetry	**t = 4.511**<br>(**p < 0.001**)<br>**t = -2.103**<br>(**p = 0.048**)
	B+CF	271.509	214.342	0.653	222.941	**F = 7.540**<br>(**p < 0.001**)	χ2 = 3.041<br>(p = 0.081)	Baseline UE-FMA score<br>Control CST FA asymmetry	**t = 4.907**<br>(**p < 0.001**)<br>t = -1.575<br>(p = 0.131)
	B+LL	198.911	162.832	0.737	215.795	**F = 11.191**<br>(**p < 0.001**)	**χ2 = 10.187**<br>(**p = 0.001**)	Baseline UE-FMA score<br>CST lesion load	**t = 4.782**<br>(**p < 0.001**)<br>**t = -3.097**<br>(**p = 0.006**)
	B+DL	193.887	161.464	0.739	215.576	**F = 11.319**<br>(**p < 0.001**)	**χ2 = 10.406**<br>(**p = 0.001**)	Baseline UE-FMA score<br>CST disconnectome load	**t = 5.844**<br>(**p < 0.001**)<br>**t = -3.137**<br>(**p = 0.005**)
	B+LV	260.637	207.406	0.665	222.086	**F = 7.926**<br>(**p < 0.001**)	χ2 = 3.896<br>(p = 0.048)	Baseline UE-FMA score<br>Lesion volume	**t = 4.648**<br>(**p < 0.001**)<br>t = -1.798<br>(p = 0.087)
	B+DV	234.815	188.181	0.696	219.557	**F = 9.144**<br>(**p < 0.001**)	**χ2 = 6.425**<br>(**p = 0.011**)	Baseline UE-FMA score<br>Disconnectome volume	**t = 5.016**<br>(**p < 0.001**)<br>**t = -2.368**<br>(**p = 0.028**)
Non-cortical	B	138.802	124.711	0.637	267.306	**F = 12.718**<br>(**p < 0.001**)	n/a	Baseline UE-FMA score	**t = 6.933**<br>(**p < 0.001**)
	PF	390.787	330.222	0.039	300.415	F = 0.291<br>(p = 0.881)	n/a	Patient CST FA asymmetry	t = 0.328<br>(p = 0.746)
	CF	373.510	316.846	0.078	299.009	F = 0.609<br>(p = 0.659)	n/a	Control CST FA asymmetry	t = 1.156<br>(p = 0.257)
	LL	374.603	327.556	0.046	300.139	F = 0.352<br>(p = 0.840)	n/a	CST lesion load	t = 0.587<br>(p = 0.562)
	DL	386.076	328.953	0.042	300.284	F = 0.320<br>(p = 0.862)	n/a	CST disconnectome load	t = 0.469<br>(p = 0.643)

Patient subgroup	Model	MSE in CV	MSE	R^2	AICc	Significance	Comparison	Predictor variable	Significance
	LV	378.670	321.246	0.065	299.477	$F = 0.502$ ($p = 0.735$)	n/a	Lesion volume	$t = 0.960$ ($p = 0.345$)
	DV	376.922	323.260	0.059	299.690	$F = 0.454$ ($p = 0.769$)	n/a	Disconnectome volume	$t = 0.857$ ($p = 0.399$)
	B+PF	148.860	128.966	0.637	270.222	**$F = 9.847$ ($p < 0.001$)**	$\chi2 = 0.052$ ($p = 0.819$)	Baseline UE-FMA score	**$t = 6.801$ ($p < 0.001$)**
								Patient CST FA asymmetry	$t = 0.208$ ($p = 0.837$)
	B+CF	150.276	127.894	0.640	269.939	**$F = 9.977$ ($p < 0.001$)**	$\chi2 = 0.336$ ($p = 0.562$)	Baseline UE-FMA score	**$t = 6.622$ ($p < 0.001$)**
								Control CST FA asymmetry	$t = -0.527$ ($p = 0.602$)
	B+LL	164.295	129.112	0.637	270.261	**$F = 9.830$ ($p < 0.001$)**	$\chi2 = 0.014$ ($p = 0.906$)	Baseline UE-FMA score	**$t = 6.751$ ($p < 0.001$)**
								CST lesion load	$t = 0.108$ ($p = 0.915$)
	B+DL	157.593	126.909	0.643	269.676	**$F = 10.098$ ($p < 0.001$)**	$\chi2 = 0.599$ ($p = 0.439$)	Baseline UE-FMA score	**$t = 6.868$ ($p < 0.001$)**
								CST disconnectome load	$t = -0.705$ ($p = 0.486$)
	B+LV	143.095	123.713	0.652	268.808	**$F = 10.503$ ($p < 0.001$)**	$\chi2 = 1.466$ ($p = 0.226$)	Baseline UE-FMA score	**$t = 6.878$ ($p < 0.001$)**
								Lesion volume	$t = -1.111$ ($p = 0.276$)
	B+DV	139.557	116.230	0.673	266.687	**$F = 11.540$ ($p < 0.001$)**	$\chi2 = 3.588$ ($p = 0.058$)	Baseline UE-FMA score	**$t = 7.256$ ($p < 0.001$)**
								Disconnectome volume	$t = -1.765$ ($p = 0.088$)

(D) Predictive models constructed via subgroup stratification according to neurophysiological status

Patient subgroup	Model	Model statistic						Predictor variable statistic	
		MSE in CV	MSE	R^2	AICc	Significance	Comparison	Predictor variable	Significance
MEP-negative	B	182.875	156.996	0.645	267.305	**$F = 12.708$ ($p < 0.001$)**	n/a	Baseline UE-FMA score	**$t = 6.670$ ($p < 0.001$)**
	PF	408.972	339.408	0.232	292.748	$F = 2.116$ ($p = 0.105$)	n/a	Patient CST FA asymmetry	**$t = -2.352$ ($p = 0.026$)**
	CF	352.120	296.076	0.330	288.241	**$F = 3.450$ ($p = 0.021$)**	n/a	Control CST FA asymmetry	**$t = -3.231$ ($p = 0.003$)**
	LL	356.011	305.380	0.309	289.262	**$F = 3.132$ ($p = 0.030$)**	n/a	CST lesion load	**$t = -3.044$ ($p = 0.005$)**
	DL	394.534	328.902	0.256	291.710	$F = 2.407$ ($p = 0.073$)	n/a	CST disconnectome load	**$t = -2.569$ ($p = 0.016$)**
	LV	390.389	337.795	0.236	292.591	$F = 2.160$ ($p = 0.100$)	n/a	Lesion volume	**$t = -2.385$ ($p = 0.024$)**
	DV	342.763	289.212	0.346	287.466	**$F = 3.699$ ($p = 0.015$)**	n/a	Disconnectome volume	**$t = -3.369$ ($p = 0.002$)**
	B+PF	186.056	156.911	0.658	269.096	**$F = 10.375$ ($p < 0.001$)**	$\chi2 = 1.218$ ($p = 0.270$)	Baseline UE-FMA score	**$t = 5.794$ ($p < 0.001$)**
								Patient CST FA asymmetry	$t = -1.008$ ($p = 0.323$)
	B+CF	172.985	144.543	0.685	266.387	**$F = 11.725$ ($p < 0.001$)**	$\chi2 = 3.927$ ($p = 0.048$)	Baseline UE-FMA score	**$t = 5.509$ ($p < 0.001$)**
								Control CST FA asymmetry	$t = -1.847$ ($p = 0.076$)
	B+LL	164.862	138.669	0.697	265.017	**$F = 12.451$ ($p < 0.001$)**	**$\chi2 = 5.296$ ($p = 0.021$)**	Baseline UE-FMA score	**$t = 5.887$ ($p < 0.001$)**
								CST lesion load	**$t = -2.168$ ($p = 0.039$)**
	B+DL	156.777	128.301	0.720	262.453	**$F = 13.893$ ($p < 0.001$)**	**$\chi2 = 7.861$ ($p = 0.005$)**	Baseline UE-FMA score	**$t = 6.692$ ($p < 0.001$)**

Patient subgroup	Model	MSE in CV	MSE	R^2	AICc	Significance	Comparison	Predictor variable	Significance
								CST disconnectome load	***t* = -2.695 (*p* = 0.012)**
	B+LV	173.181	148.068	0.677	267.182	***F* = 11.317 (*p* < 0.001)**	χ2 = 3.132 (*p* = 0.077)	Baseline UE-FMA score<br>Lesion volume	***t* = 6.073 (*p* < 0.001)**<br>*t* = -1.640 (*p* = 0.113)
	B+DV	158.376	131.565	0.713	263.282	***F* = 13.414 (*p* < 0.001)**	**χ2 = 7.032 (*p* = 0.008)**	Baseline UE-FMA score<br>Disconnectome volume	***t* = 5.878 (*p* < 0.001)**<br>***t* = -2.532 (*p* = 0.017)**
MEP-positive	B	11.157	8.168	0.926	80.448	***F* = 28.235 (*p* < 0.001)**	n/a	Baseline UE-FMA score	***t* = 8.594 (*p* < 0.001)**
	PF	88.203	50.226	0.546	105.876	*F* = 2.708 (*p* = 0.099)	n/a	Patient CST FA asymmetry	*t* = 2.115 (*p* = 0.064)
	CF	58.260	30.964	0.720	99.104	***F* = 5.792 (*p* = 0.014)**	n/a	Control CST FA asymmetry	***t* = 3.586 (*p* = 0.006)**
	LL	72.510	40.360	0.635	102.814	***F* = 3.920 (*p* = 0.041)**	n/a	CST lesion load	***t* = 2.787 (*p* = 0.021)**
	DL	46.349	29.278	0.735	98.320	***F* = 6.255 (*p* = 0.011)**	n/a	CST disconnectome load	***t* = 3.757 (*p* = 0.005)**
	LV	149.671	67.141	0.393	109.940	*F* = 1.459 (*p* = 0.292)	n/a	Lesion volume	*t* = 1.039 (*p* = 0.326)
	DV	30.058	21.719	0.804	94.139	***F* = 9.215 (*p* = 0.003)**	n/a	Disconnectome volume	***t* = 4.707 (*p* = 0.001)**
	B+PF	10.296	7.194	0.942	83.520	***F* = 26.091 (*p* < 0.001)**	χ2 = 3.427 (*p* = 0.064)	Baseline UE-FMA score<br>Patient CST FA asymmetry	***t* = 7.405 (*p* < 0.001)**<br>*t* = 1.490 (*p* = 0.175)
	B+CF	15.459	8.436	0.932	85.750	***F* = 22.014 (*p* < 0.001)**	χ2 = 1.197 (*p* = 0.274)	Baseline UE-FMA score<br>Control CST FA asymmetry	***t* = 5.003 (*p* = 0.001)**<br>*t* = 0.845 (*p* = 0.423)
	B+LL	16.295	8.885	0.929	86.476	***F* = 20.820 (*p* < 0.001)**	χ2 = 0.471 (*p* = 0.492)	Baseline UE-FMA score<br>CST lesion load	***t* = 5.734 (*p* < 0.001)**<br>*t* = 0.523 (*p* = 0.615)
	B+DL	15.879	8.824	0.929	86.380	***F* = 20.976 (*p* < 0.001)**	χ2 = 0.568 (*p* = 0.451)	Baseline UE-FMA score<br>CST disconnectome load	***t* = 4.676 (*p* = 0.002)**<br>*t* = 0.575 (*p* = 0.581)
	B+LV	18.112	8.145	0.935	85.260	***F* = 22.856 (*p* < 0.001)**	χ2 = 1.688 (*p* = 0.194)	Baseline UE-FMA score<br>Lesion volume	***t* = 8.136 (*p* < 0.001)**<br>*t* = 1.013 (*p* = 0.341)
	B+DV	9.398	6.470	0.948	82.036	***F* = 29.188 (*p* < 0.001)**	χ2 = 4.911 (*p* = 0.027)	Baseline UE-FMA score<br>Disconnectome volume	***t* = 4.713 (*p* = 0.002)**<br>*t* = 1.833 (*p* = 0.104)

(E) Predictive models constructed via subgroup stratification according to proportional recovery

**Patient subgroup**	**Model**	**Model statistic**						**Predictor variable statistic**	
		**MSE in CV**	**MSE**	R^2	**AICc**	**Significance**	**Comparison**	**Predictor variable**	**Significance**
Non-fitted	B	51.358	41.221	0.543	152.328	***F* = 5.047 (*p* = 0.007)**	n/a	Baseline UE-FMA score	***t* = 4.042 (*p* = 0.001)**
	PF	58.497	44.247	0.509	153.886	***F* = 4.411 (*p* = 0.013)**	n/a	Patient CST FA asymmetry	***t* = -3.749 (*p* = 0.002)**
	CF	60.444	47.164	0.477	155.291	***F* = 3.876 (*p* = 0.020)**	n/a	Control CST FA asymmetry	***t* = -3.484 (*p* = 0.003)**
	LL	55.640	45.216	0.499	154.363	***F* = 4.226 (*p* = 0.015)**	n/a	CST lesion load	***t* = -3.660 (*p* = 0.002)**

	DL	76.807	59.154	0.344	160.274	F = 2.229 (p = 0.109)	n/a	CST disconnectome load	**t = -2.496 (p = 0.023)**
	LV	84.442	62.173	0.311	161.369	F = 1.914 (p = 0.154)	n/a	Lesion volume	**t = -2.259 (p = 0.037)**
	DV	57.995	48.780	0.459	156.032	**F = 3.606 (p = 0.026)**	n/a	Disconnectome volume	**t = -3.342 (p = 0.004)**
	B+PF	43.753	31.732	0.669	149.088	**F = 6.462 (p = 0.002)**	**$\chi2$ = 7.089 (p = 0.008)**	Baseline UE-FMA score<br>Patient CST FA asymmetry	**t = 2.776 (p = 0.013)**<br>**t = -2.466 (p = 0.025)**
	B+CF	45.571	32.872	0.657	149.865	**F = 6.127 (p = 0.002)**	**$\chi2$ = 6.312 (p = 0.012)**	Baseline UE-FMA score<br>Control CST FA asymmetry	**t = 2.897 (p = 0.011)**<br>**t = -2.306 (p = 0.035)**
	B+LL	45.847	33.812	0.647	150.485	**F = 5.868 (p = 0.003)**	**$\chi2$ = 5.693 (p = 0.017)**	Baseline UE-FMA score<br>CST lesion load	**t = 2.595 (p = 0.020)**<br>**t = -2.174 (p = 0.045)**
	B+DL	48.740	35.383	0.631	151.484	**F = 5.465 (p = 0.004)**	**$\chi2$ = 4.693 (p = 0.030)**	Baseline UE-FMA score<br>CST disconnectome load	**t = 3.524 (p = 0.003)**<br>t = -1.951 (p = 0.069)
	B+LV	51.212	39.338	0.589	153.815	**F = 4.594 (p = 0.009)**	$\chi2$ = 2.362 (p = 0.124)	Baseline UE-FMA score<br>Lesion volume	**t = 3.297 (p = 0.005)**<br>t = -1.347 (p = 0.197)
	B+DV	46.618	34.003	0.645	150.609	**F = 5.817 (p = 0.003)**	**$\chi2$ = 5.568 (p = 0.018)**	Baseline UE-FMA score<br>Disconnectome volume	**t = 2.896 (p = 0.011)**<br>**t = -2.147 (p = 0.047)**
	B	47.986	41.279	0.597	255.727	**F = 12.218 (p < 0.001)**	n/a	Baseline UE-FMA score	**t = 6.570 (p < 0.001)**
	PF	111.021	95.256	0.070	287.503	F = 0.620 (p = 0.652)	n/a	Patient CST FA asymmetry	t = -0.062 (p = 0.951)
	CF	112.491	95.182	0.071	287.474	F = 0.627 (p = 0.647)	n/a	Control CST FA asymmetry	t = 0.171 (p = 0.865)
	LL	110.326	94.784	0.074	287.314	F = 0.664 (p = 0.622)	n/a	CST lesion load	t = -0.410 (p = 0.684)
	DL	110.870	95.262	0.070	287.505	F = 0.619 (p = 0.652)	n/a	CST disconnectome load	t = 0.043 (p = 0.966)
	LV	107.915	93.985	0.082	286.992	F = 0.740 (p = 0.572)	n/a	Lesion volume	t = -0.671 (p = 0.507)
	DV	111.305	95.201	0.070	287.481	F = 0.625 (p = 0.648)	n/a	Disconnectome volume	t = -0.152 (p = 0.880)
Fitted	B+PF	51.507	42.564	0.597	258.557	**F = 9.480 (p < 0.001)**	$\chi2$ = 0.005 (p = 0.945)	Baseline UE-FMA score<br>Patient CST FA asymmetry	**t = 6.469 (p < 0.001)**<br>t = 0.063 (p = 0.950)
	B+CF	52.169	42.491	0.598	258.492	**F = 9.507 (p < 0.001)**	$\chi2$ = 0.069 (p = 0.792)	Baseline UE-FMA score<br>Control CST FA asymmetry	**t = 6.475 (p < 0.001)**<br>t = -0.242 (p = 0.811)
	B+LL	51.093	42.126	0.601	258.164	**F = 9.645 (p < 0.001)**	$\chi2$ = 0.397 (p = 0.529)	Baseline UE-FMA score<br>CST lesion load	**t = 6.500 (p < 0.001)**<br>t = -0.580 (p = 0.566)
	B+DL	49.569	41.073	0.611	257.202	**F = 10.056 (p < 0.001)**	$\chi2$ = 1.359 (p = 0.244)	Baseline UE-FMA score<br>CST disconnectome load	**t = 6.674 (p < 0.001)**<br>t = -1.079 (p = 0.288)
	B+LV	50.569	42.423	0.598	258.431	**F = 9.533 (p < 0.001)**	$\chi2$ = 0.131 (p = 0.717)	Baseline UE-FMA score	**t = 6.412 (p < 0.001)**

								Lesion volume	$t = -0.332$ ($p = 0.742$)
	B+DV	52.148	42.300	0.599	258.321	**$F = 9.579$ ($p < 0.001$)**	$\chi 2 = 0.241$ ($p = 0.624$)	Baseline UE-FMA score	**$t = 6.502$ ($p < 0.001$)**
								Disconnectome volume	$t = -0.451$ ($p = 0.655$)

CV, cross-validation; UE-FMA, Upper extremity Fugl-Meyer assessment; CST, corticospinal tract; and FA, fractional anisotropy.

Table S4. Comparison table of the goodness of fit between predictive models developed for the whole group (A) and for stratified subgroups (B-E). Comparisons were made for predictive models comprising the combination of baseline upper limb motor impairment and lesion-induced brain structural damage. The labels of the models refer to those listed in Table S2.

(A) Predictive models constructed for the whole group

**Subgroup**	**Model**	B+PF	B+CF	B+LL	B+DL	B+LV	B+DV
All individuals	B+PF						
	B+CF	p = 1.000					
	B+LL	p = 0.831	p = 0.831				
	B+DL	p = 0.839	p = 0.839	p = 0.992			
	B+LV	p = 1.000	p = 1.000	p = 0.831	p = 0.839		
	B+DV	p = 0.939	p = 0.939	p = 0.891	p = 0.899	p = 0.939	

(B) Predictive models constructed via subgroup stratification according to initial impairment

**Patient subgroup**	**Model**	B+PF	B+CF	B+LL	B+DL	B+LV	B+DV
Severe	B+PF						
	B+CF	p = 0.909					
	B+LL	p = 0.926	p = 0.836				
	B+DL	p = 0.811	p = 0.723	p = 0.883			
	B+LV	p = 0.830	p = 0.921	p = 0.759	p = 0.650		
	B+DV	p = 0.909	p = 1.000	p = 0.836	p = 0.723	p = 0.921	
Non-severe	B+PF						
	B+CF	p = 0.872					
	B+LL	p = 0.864	p = 0.993				
	B+DL	p = 0.822	p = 0.949	p = 0.956			
	B+LV	p = 0.822	p = 0.949	p = 0.956	p = 1.000		
	B+DV	p = 0.822	p = 0.949	p = 0.956	p = 1.000	p = 1.000	

(C) Predictive models constructed via subgroup stratification according to lesion location

**Patient subgroup**	**Model**	B+PF	B+CF	B+LL	B+DL	B+LV	B+DV
Cortical	B+PF						
	B+CF	p = 0.861					
	B+LL	p = 0.698	p = 0.573				
	B+DL	p = 0.686	p = 0.563	p = 0.988			
	B+LV	p = 0.919	p = 0.941	p = 0.624	p = 0.614		
	B+DV	p = 0.922	p = 0.785	p = 0.772	p = 0.760	p = 0.842	
Non-cortical	B+PF						
	B+CF	p = 0.984					
	B+LL	p = 1.000	p = 0.984				
	B+DL	p = 0.967	p = 0.984	p = 0.967			
	B+LV	p = 0.918	p = 0.934	p = 0.918	p = 0.950		
	B+DV	p = 0.799	p = 0.815	p = 0.799	p = 0.831	p = 0.880	

(D) Predictive models constructed via subgroup stratification according to neurophysiological status

**Patient subgroup**	**Model**	B+PF	B+CF	B+LL	B+DL	B+LV	B+DV
MEP-negative	B+PF						
	B+CF	*p* = 0.906					
	B+LL	*p* = 0.863	*p* = 0.956				
	B+DL	*p* = 0.778	*p* = 0.869	*p* = 0.912			
	B+LV	*p* = 0.935	*p* = 0.972	*p* = 0.928	*p* = 0.841		
	B+DV	*p* = 0.804	*p* = 0.896	*p* = 0.940	*p* = 0.973	*p* = 0.868	
MEP-positive	B+PF						
	B+CF	*p* = 0.750					
	B+LL	*p* = 0.686	*p* = 0.931				
	B+DL	*p* = 0.686	*p* = 0.931	*p* = 1.000			
	B+LV	*p* = 0.820	*p* = 0.928	*p* = 0.859	*p* = 0.859		
	B+DV	*p* = 0.828	*p* = 0.592	*p* = 0.534	*p* = 0.534	*p* = 0.656	

(E) Predictive models constructed via subgroup stratification according to proportional recovery

**Patient subgroup**	**Model**	B+PF	B+CF	B+LL	B+DL	B+LV	B+DV
Non-fitted	B+PF						
	B+CF	*p* = 0.946					
	B+LL	*p* = 0.903	*p* = 0.956				
	B+DL	*p* = 0.835	*p* = 0.888	*p* = 0.932			
	B+LV	*p* = 0.674	*p* = 0.724	*p* = 0.766	*p* = 0.832		
	B+DV	*p* = 0.894	*p* = 0.948	*p* = 0.991	*p* = 0.941	*p* = 0.774	
Fitted	B+PF						
	B+CF	*p* = 0.995					
	B+LL	*p* = 0.978	*p* = 0.984				
	B+DL	*p* = 0.924	*p* = 0.930	*p* = 0.946			
	B+LV	*p* = 0.995	*p* = 1.000	*p* = 0.984	*p* = 0.930		
	B+DV	*p* = 0.989	*p* = 0.995	*p* = 0.989	*p* = 0.935	*p* = 0.995	

MEP, motor evoked potential.

Supplementary Figures

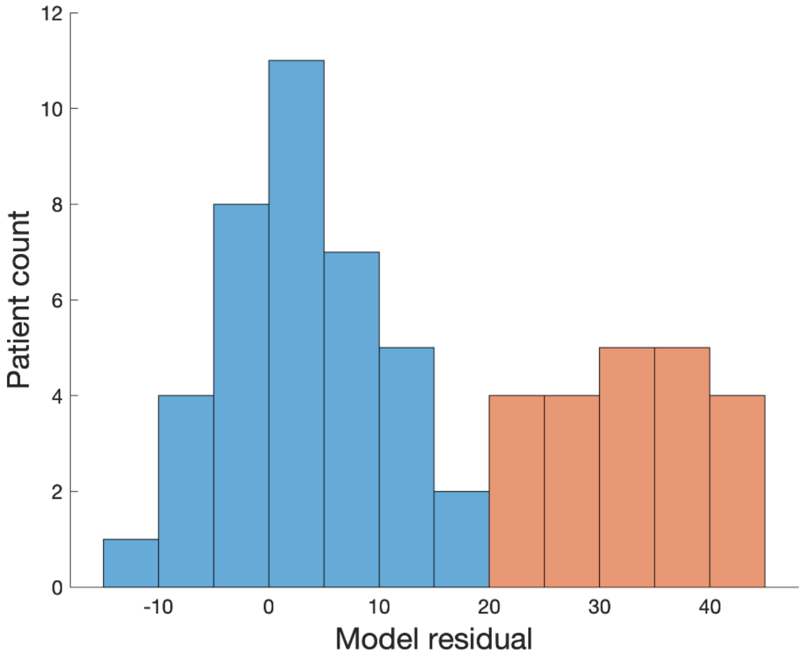

Fig. S1. Histogram of proportional recovery model residuals. Model residuals were measured by the difference between the predicted change in the upper extremity Fugl-Meyer assessment (UE-FMA) score according to the proportional recovery rule and the observed change in the UE-FMA score. At the threshold of the model residual of 20, individuals with stroke were allocated to non-fitted and fitted subgroups, indicated as red and blue bars, respectively.

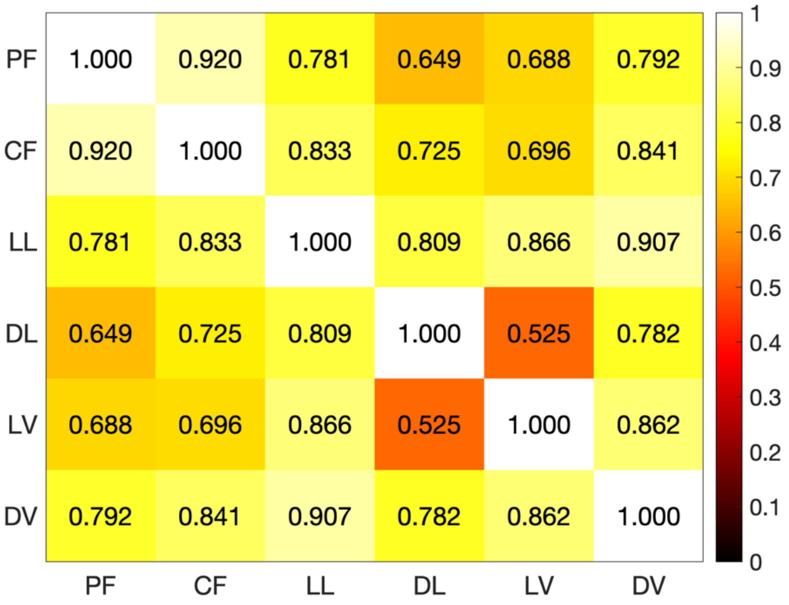

Fig. S2. Correlation map of brain structural damage measures. The red-yellow colour maps the pair-wise correlation coefficient. PF, patient CST FA asymmetry; CF, control CST FA asymmetry; LL, CST lesion load; DL, CST disconnectome load; LV, lesion volume; and DV, disconnectome volume.
